# Chloroform in Strangulated Hernia

**Published:** 1854-08

**Authors:** B. F. Taylor

**Affiliations:** Louisiana


CHLOROFORM IN STRANGULATED HERNIA.—BY B. F. TAYLOR, M. D., of Louisiana.—Of all the maladies to which the human body is subject, that of irreducible hernia is probably the most formidable and important, calling forth the greatest amount of surgical and anatomical knowledge, requiring the most prompt and decisive action, as well as the utmost skill and dexterity in the performance of an operation, when that is rendered necessary by the inefficacy of the means usually employed to accomplish its reduction. In most surgical diseases, the patient can have the advantage of consultations with the most talented and experienced of the faculty, but not unfrequently an hour's delay will prove prejudicial to the success of the surgeon and fatal to the prospects of the patient.

It is known to practical surgeons, that the best directed efforts of the taxis, the use of cathartics, opium, bleeding and tobacco injections, not unfrequently fail in relaxing the strictured parts. In view of these difficulties, it has often occurred to me—since the introduction of chloroform as an anæsthetic agent in surgical practice—that the full influence of this article would fulfil the indications above spoken of, and probably dispense with—in the majority of cases—this formidable operation.

A case in point occurred recently, in my practice, where I had an opportunity of testing the correctness of these views. The patient, a middle aged man, had been afflicted with a hernia on the left side for a considerable length of time. Upon one occasion, when employed in wood-chopping, the hernia became strangulated; when I was sent for, and reduced it readily by the taxis. More recently, this man being engaged in the field with his hoe, strangulation again occurred. Upon visiting him I found his suffering to be very intense; pain in the abdomen and stomach, vomiting, &c. The tumor was very large and tender to the touch. The stricture, as is most generally the case in inguinal hernias of long standing, was situated at the external abdominal ring. The taxis was resorted to, and persevered in, but without beneficial results. He was then put under the full effect of chloroform. Relaxation of the external oblique muscle, and the stricture at the ring, was evident to the touch. The taxis was again resorted to, when the gut readily returned with a distinct gurgling sound.

This case is reported for the sole purpose of calling the attention of surgeons to the practical value and use of this most potent of remedies. It is an isolated fact, it is true; but in all cases where relaxation of the voluntary muscles is the object to be obtained—such as luxations, hour glass contractions of the uterus, &c.—we are strongly convinced that the practioner's most sanguine hopes will be more than fully realized.

